# Blended learning in quality improvement training for healthcare professionals in Qatar

**DOI:** 10.5116/ijme.5a80.3d88

**Published:** 2018-02-23

**Authors:** Shireen Suliman, Reham Hassan, Khawla Athamneh, Marjorie Jenkins, Carma Bylund

**Affiliations:** 1 Department of Internal Medicine, Hamad Medical Corporation, Doha, Qatar; 2 Johns Hopkins University School of Education, USA

**Keywords:** Blended learning, quality improvement training, healthcare professionals, Qatar

## Introduction

Training healthcare providers (HCP) in quality improvement (QI) is essential for both the HCP and the organization. Physicians and others who work in QI have developed the habit of being concerned with better patient outcomes, better system performance and better professional development as separate and distinct entities.^1^ During the last five years, the Clinical Care Improvement Training Program (CCITP) has been implemented as a formal curriculum that teaches QI principles to health care providers across Hamad Medical Corporation (HMC), a large JCI accredited group of tertiary hospitals in Qatar. Education of these health care providers occurs in the form of face-to-face didactic lectures. In addition, participants work collaboratively in groups to apply their knowledge in designing and implementing QI projects at their departments.

While lectures are powerful methods for delivering large amount of information, studies have shown that interactive teaching styles are more popular and that the use of more of them would result in better knowledge retention.^2^^,^^3^ Thus, through combining them with other modalities such as simulation, problem based learning and group discussions, a better learning experience is achieved.^4^^, ^^5^

E-learning embraces an approach that typically aspires to be flexible, engaging and learner–centered; one that encourages interaction, and collaboration and communication, often asynchronously.^6^ Blended learning (BL), also known as hybrid learning, is a concept that describes the integration of classroom face-to-face instructions with online learning experiences. BL facilitates asynchronous collaborative learning together with the pedagogical self-directed learning provided in the online platform while keeping the socialization benefit of face-to-face learning. Knowledge is continuously changing and advancing in medicine, therefore encouraging self-directed learning through e-learning would result in long term retention of knowledge unlike the passive absorption of knowledge that occurs in didactic lectures. The purpose of this paper is to demonstrate the successful implementation process of BL in teaching quality to HCP in Qatar and to report on the participants’ evaluation.

### Program description

The CCITP is a program that was developed in 2011 at HMC in Qatar. It runs in two four-month-cycles per year, with the number of participants ranging from 40 to 60 each cycle.  Participants receive training through traditional face-to-face teaching facilitated by SharePoint, which was used to upload assignments (web-based). All these participants are HMC healthcare providers from different disciplines and hospitals selected by their departments to be enrolled in the CCITP. They are physicians (including consultants, fellows and residents), nurses, and other allied healthcare professionals (including paramedics and pharmacists), and quality reviewers.

The BL format was introduced into the CCITP in January 2016, but the planning process started 6 months earlier. Engagement of many stakeholders was a requisite for the implementation process. With the assistance of the dedicated IT department personnel at HMC, we incorporated the new CCITP online platform into the corporate intranet with remote access and the ability to accommodate all the materials. This was followed by preparation and delivery of an orientation video for the faculty, coaches and participants demonstrating the access and use of the workspace. Developing an assortment of online materials was the next important step. Those materials complemented face-to-face lectures and were designed to provide rich resources for carrying out the activities and answering the discussion questions. A committee of experts in quality reviewed these materials thoroughly during each module before being uploaded into the workspace. Beside the resources, we created online group activities to facilitate learning of quality improvement tools. We added a discussion board to the workspace to enhance learning through communication. There were continuous project improvement efforts to upgrade the workspace, to solve technical issues and change its look to be more attractive and accessible to the participants. Two workspace orientation sessions were arranged and conducted with the help of one of the IT personnel and the CCITP faculty during the cycle. At the midpoint of the project, a feedback discussion was conducted with the coaches and faculty to identify any challenges with the use of the workspace. Over a course of four months, CCITP participants continued to learn through the monthly face to face didactic lectures and getting the benefit of the online materials to work cooperatively on the online activities and assignments.

At the end of the cycle, we used an online satisfaction survey to evaluate the program. There was an overall perceived improvement in both knowledge and skills of the participants. Participants found that the requirements and deadlines of the course were clear, the various aspects of the course (lectures, discussions, readings, online activities) were integrated into a coherent whole and the course created a sense of community among participants.

Many factors contributed to high satisfaction of the BL among participants.

  1.   Coherence in BL where the majority of the participants valued the course goals and objectives as being clear and the various aspects of the course including the face-to-face and online discussions, readings, and activities as being integrated into a coherent whole to the extent that they would recommend this type of hybrid class to others. Our results are comparable to Garrison & Kanuka’s study that has shown that BL has the proven transformational potential to enhance both the effectiveness and efficiency of meaningful learning experiences.^7^

  2.   Pedagogy in BL where participants indicated that the BL experience has increased their opportunity to have convenient access to and use of information and the online component of the course created an opportunity to learn outside the classroom, through engaging in discussions, activities and assignments. Participants valued course design containing options, personalization, self-direction, variety and a learning community.^8^

  3.   Collaboration in BL where CCITP participants reported that the BL has enhanced learning and their quality improvement skills through information sharing where they have to work on the online activities and assignments in groups. Enhancing participants’ interaction in group activities has a positive impact on the learning process unlike the didactic lectures.^9^ The new BL format created a sense of community among participants through group discussion and collaboration and thus produced a stronger sense of community among students than either traditional or fully online courses through providing collaborative learning experiences.^10^

## Conclusions

BL is a well-perceived teaching modality in medical education. It can be implemented successfully in a quality training program to enhance learners’ knowledge in quality improvement science. Leadership support was a key element in the successful implementation of BL together with engagement of the faculty and coaches. Communicating the vision through open discussion with CCITP faculty and coaches facilitated their acceptance of the change, their participation and commitment. Participants had more time to review the course materials at their own time. Through establishing coherence between the in-class and the online learning together with facilitating collaboration and group work in online assignments and activities, we can improve participants’ knowledge and satisfaction in learning about quality improvement science.

### Conflict of Interest

The authors declare that they have no conflict of interest.
